# Supplementary material for: Inhibition of DNA methylation promotes breast tumor sensitivity to netrin‐1 interference
Source: EMBO Mol Med. 2016 Jul 4;8(8):863–77. doi: 10.15252/emmm.201505945 (PMC4967941; doi:10.15252/emmm.201505945)
Supplement: Supplementary file 1 — Appendix [file EMMM-8-863-s001.pdf]

Inhibition of DNA methylation promotes tumor sensitivity to netrin-1 interference.

Mélodie Grandin[1], Pauline Mathot[1], Guillaume Devailly[1], Yannick Bidet[2], Akram Ghantous[3], Clementine Favrot[1], Benjamin Gibert[1], Nicolas Gadot[4], Isabelle Puisieux[5], Zdenko Herceg[3], Jean-Guy Delcros[1], Agnès Bernet[1], Patrick Mehlen[1$], Robert Dante[1$]

Appendix Figure S1 is related to Figure 3.

Appendix Figure S2 is related to Figure 3.

Appendix Figure S3 is related to Figures 3 and 4.

Appendix Figure S4 is related to Figures 3 and 4.

Appendix Figure S5 is related to Figure 5.

Appendix Figure S6 is related to Figure 5.

Appendix Table S1 is related to Experimental Procedures

Appendix Figure S1 - The netrin-1 blocking antibody net1-mAb triggered apoptotic cell death in breast cancer cell lines upon 5-azacytidine treatment.

A, B Gene expression was measured by Q-RT-PCR after 72 hr in MDA-MB-231 (A) and HMLER (B) cells treated daily with 2 µM of 5-azacytidine (Aza). *PBGD* expression level was used as internal control. Error bars, s.e.m of at least 3 independent experiments, two-tailed unpaired Student's t test, ns = not significant.

C, F MDA-MB-231 and HMLER cell lines were treated with Aza, 2 µM for 72h, and/or net1-mAb (10 µg/mL, 48h) and/or recombinant netrin-1 (5 µg/mL, 48h). Caspase-3 activities (C and E) and cellular mortality (D and F) were determined in 3 independent experiments. **** P<0.0001. ANOVA1. Error bars = s.e.m

Appendix Figure S2 – Tumor growth inhibition induced by treatment combining DAC and therapeutic netrin-1 antibody, *in vivo*.

A. When tumors reached 100 mm^3, mice were injected subcutaneously with decitabine (0.5mg/kg) or PBS and/or intraperitoneally with NET1-mAb-T (10 mg/kg) or a Control IgG (Iso-mAb (10 mg/kg). Tumor volumes were measured twice a week. Statistical significance of differences between PBS-groups and DAC + NET1-mAb-T-groups was determined by two-way ANOVA and post-hoc Tukey-test. (n = 6 mice per group). **** P<0.0001.

B. Activated caspase-3, median number of cells per mm^2 were measured using antibody against cleaved caspase-3 and the DNA fragmentation median number of cells per mm^2 were determined by TUNEL assay (B); mean values from 4 tumors per group. **** P<0.0001, ANOVA1

C. Representative tumor sections corresponding to the histograms (B), bars represent 50 µm.

Appendix Figure S3 – Angiogenesis and cell proliferation in patient-derived xenografts treated net1-mAb and/ or decitabine (DAC).

A, D Paraffin embedded tumor xenografts were stained using CD31 for angiogenesis and Ki-67 for cell proliferation in MDA-MB-231 (A, B) and PDX HBC 146 (C, D).

(A and C) Vessel numbers in tumors were measured by CD31 immunohistochemistry staining and expressed as median number of vessels per mm^2

(A and C) Mean proliferation rate assessed by Ki- 67 staining; proliferative cells were divided by non-proliferative cells. Error bars = s.e.m. ANOVA1; *** P<0.001, n = not significant, ANOVA1

(B and D) Representative sections of CD31 and KI67 staining for angiogenesis and proliferation count.

Appendix Figure S4 - Combined treatment led to a reduced number of metastasis formation, and was not associated with changes in angiogenesis or proliferation.

A, B. MDA-MB-231 cells previously treated with control IgG1, DAC and/or net1-mAb alone or in combination were grafted on the chorio-allantoic membrane (CAM) of 10 days old chicken embryos. Error bars = s.e.m, Kruskal–Wallis test.

Appendix Figure S5 - Genes upregulated by decitabine treatment in MDA-MB231 and HMLER cell lines.

A. Venn diagram showing the overlap of genes upregulated by decitabine treatment in MDA-MB-231 and HMLER cell lines.

B. Gene ontology and KEGG pathways identified from of common DAC-upregulated genes between MDA-MB-231 and HMLER cell lines.

Appendix Figure S6 – Expression of genes involved in the YAP signaling pathway and effect of *IRF7* depletion on *DAPK1*, *UNC5B*, and *NTN1* expression.

A, B Q-RT-PCR assays of the expression of genes linked to the netrin-1 dependence receptor pathway, *DAPK1*, *UNC5B*, and *NTN1*, and genes linked to the YAP signaling pathway, *YAP*, *TAZ*, and *CTGF* in MDA-MB-231 (A) and HMLER (B) cells. Error bars = s.e.m. *** P<0.001, ns = not significant. ANOVA1

C, D Q-RT-PCR assays of the expression of *DAPK1*, *UNC5B*, *NTN1*, and *IRF7* upon treatment combining DAC treatment and transient transfection with a siRNA targeting *iRF7* in MDA-MB-231 (C) and HMLER (D) cells. **** P<0.0001, ns = not significant. ANOVA1.

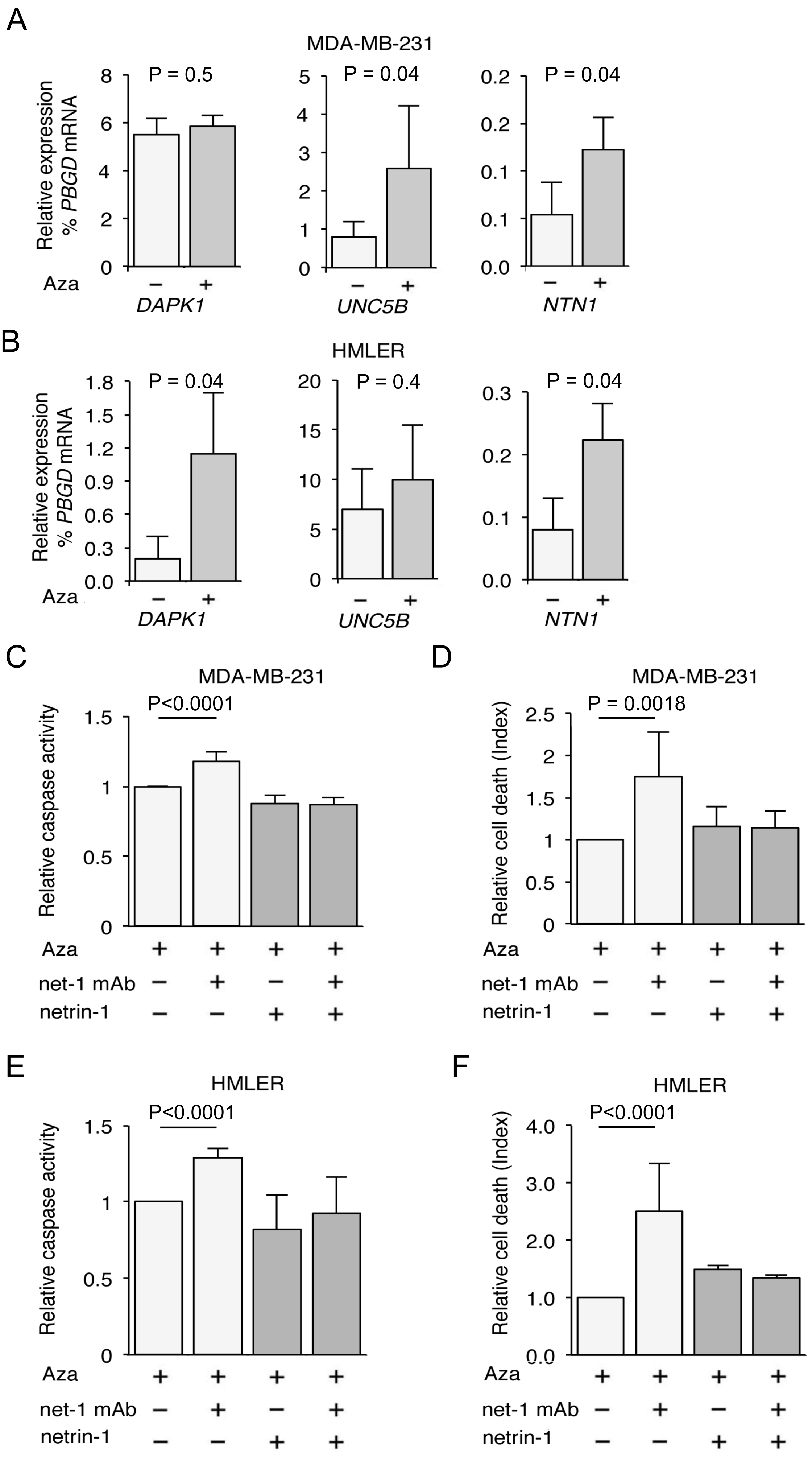

Appendix Figure S1. Grandin et al.

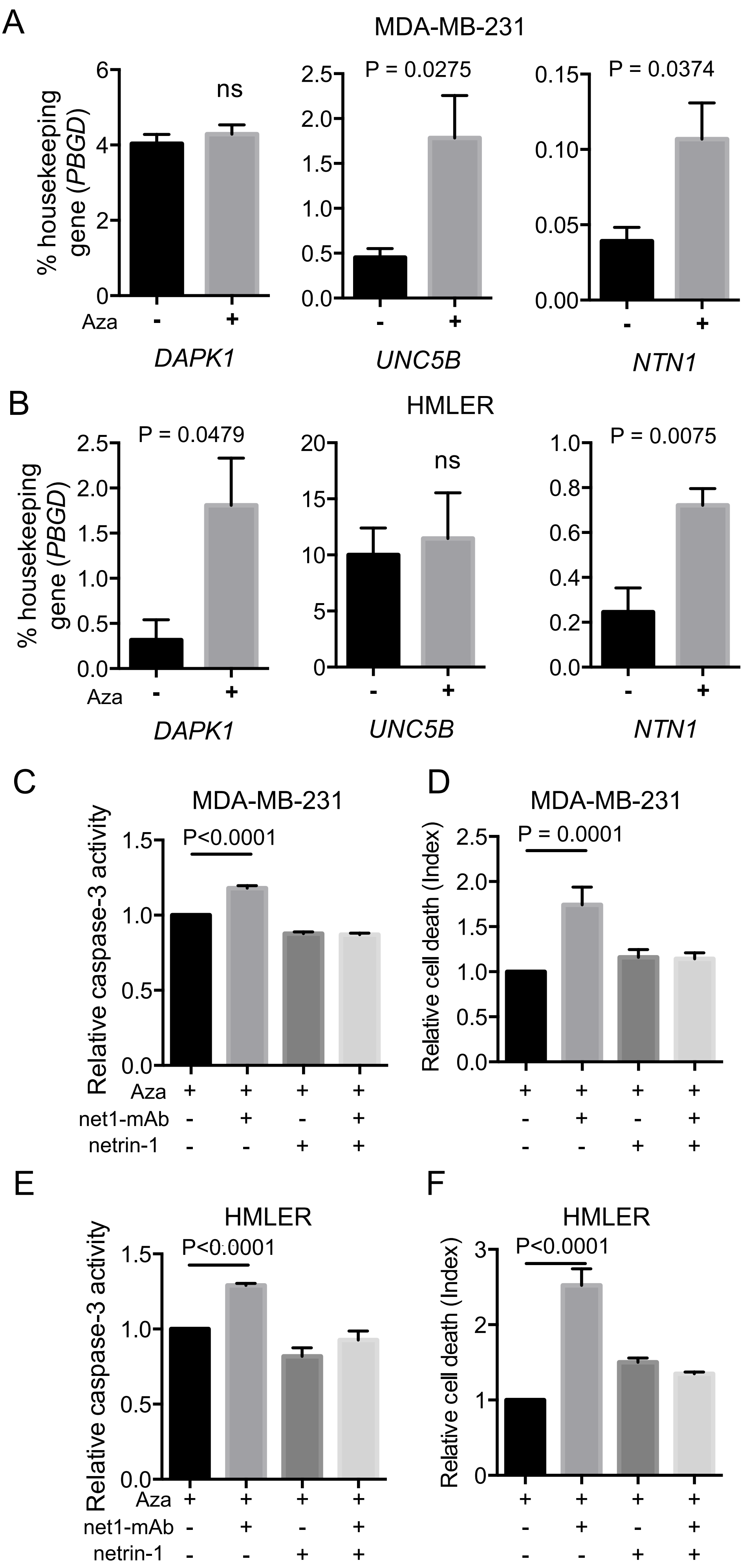

Appendix Figure S1. Grandin et al.

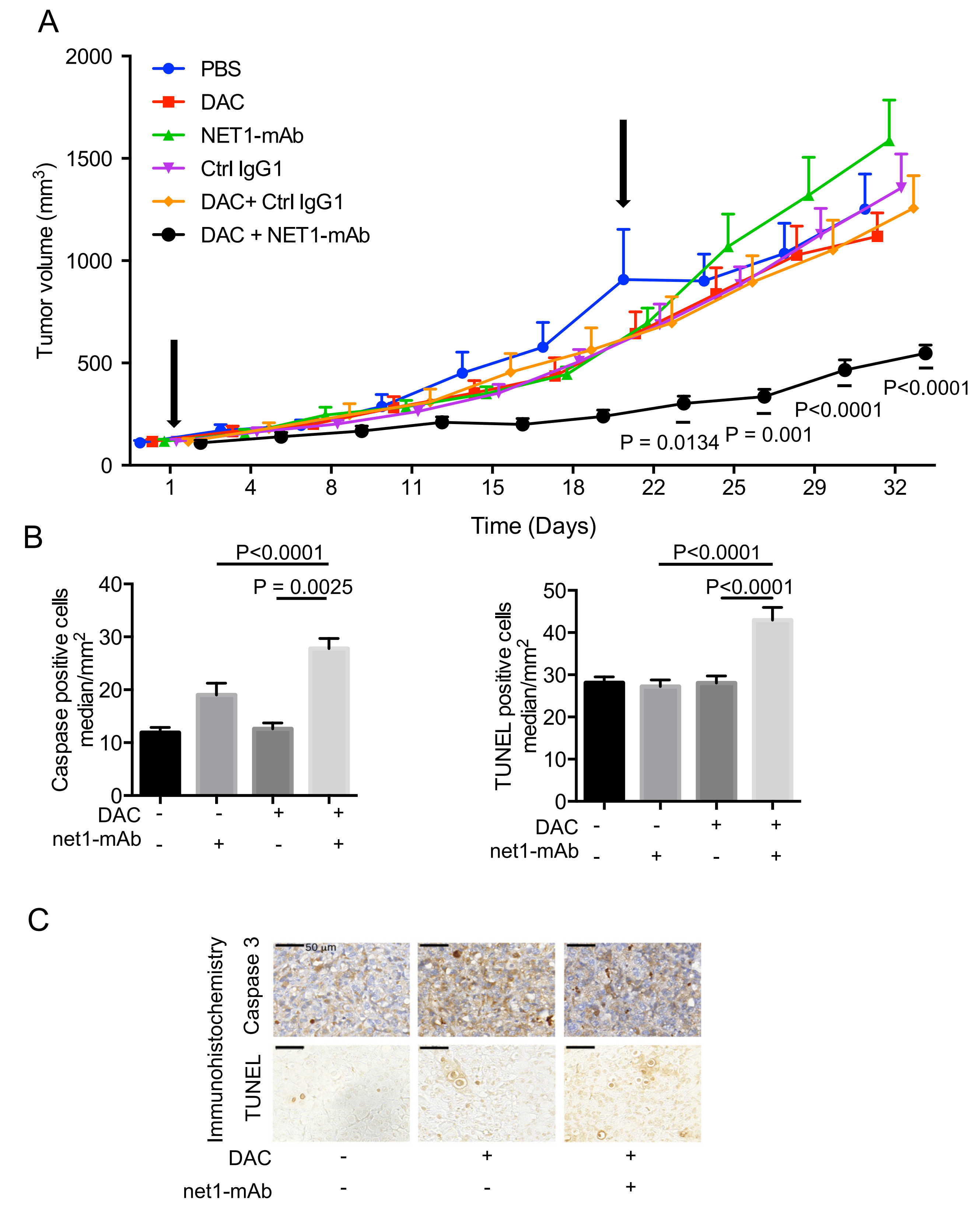

Appendix Figure S2. Grandin et al.

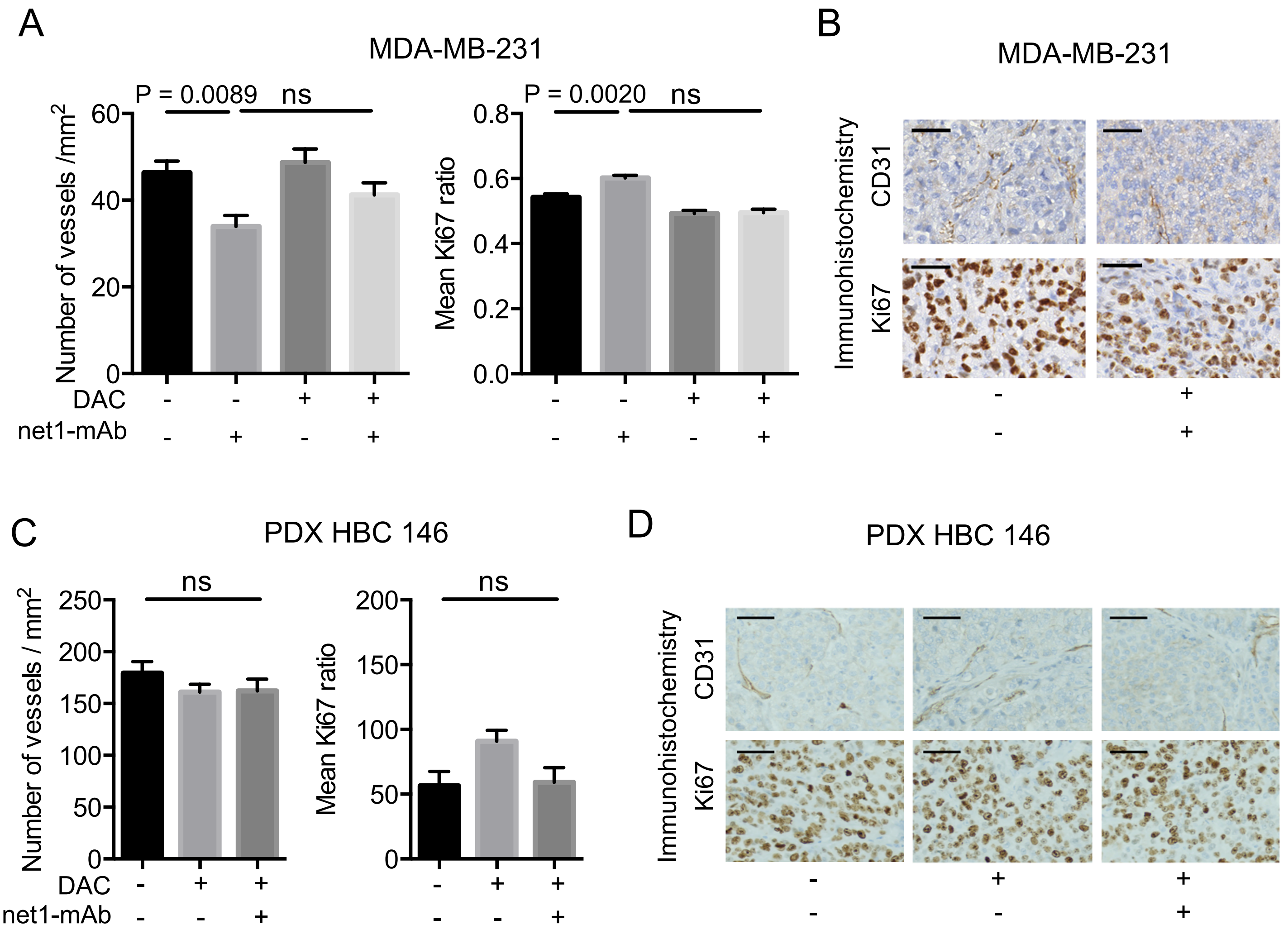

Appendix Figure S3. Grandin et al.

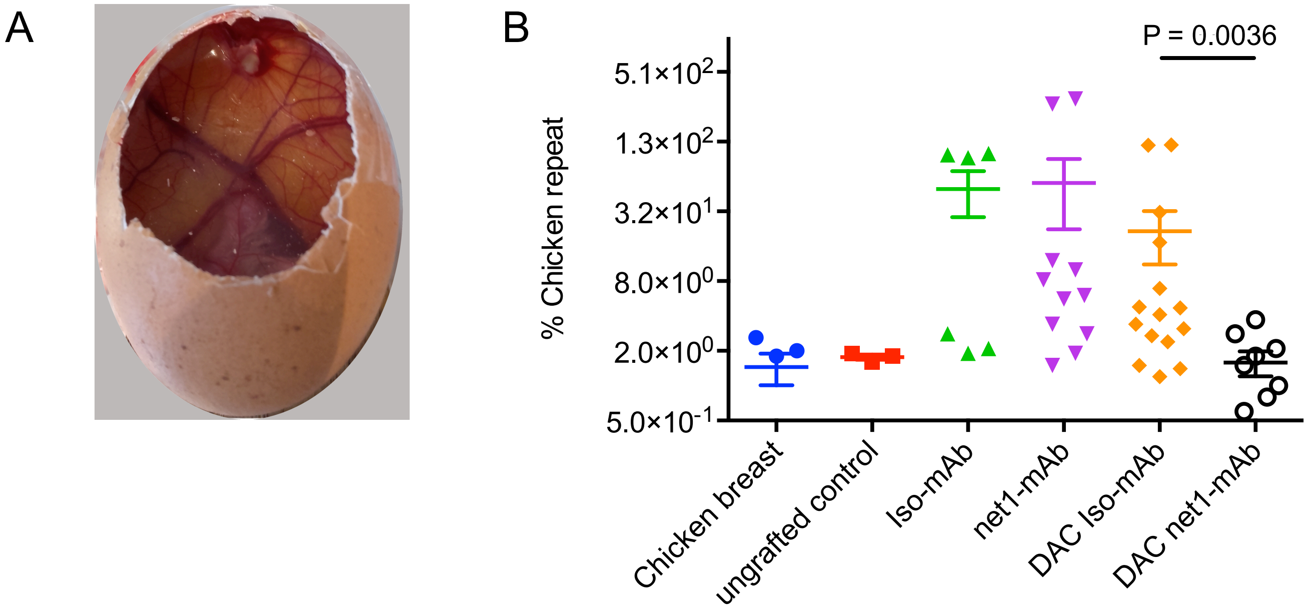

Appendix Figure S4. Grandin et al.

A

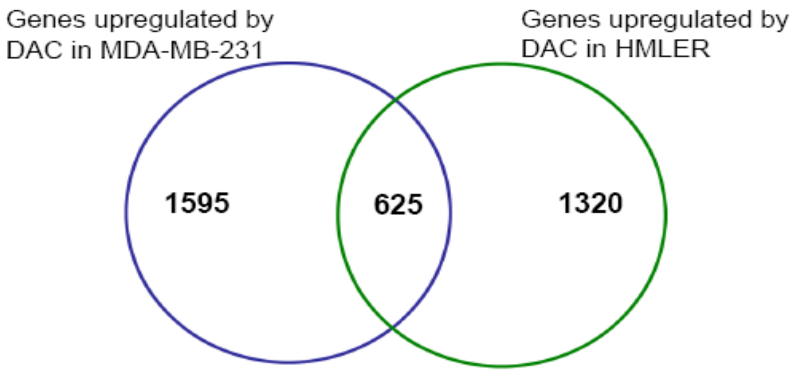

B

Set	Rank	Name	p value	ajusted p value
KEGG pathways	1	Osteoclast differentiation	2.40E-07	1.42E-05
	2	MAPK signaling pathway	7.50E-06	8.85E-05
	3	Rheumatoid arthritis	5.86E-06	8.85E-05
	4	Cytokine-cytokine receptor interaction	6.85E-06	8.85E-05
	5	Pathways in cancer	5.18E-06	8.85E-05
	6	NOD-like receptor signaling pathway	9.69E-06	9.53E-05
	7	African trypanosomiasis	2.54E-05	2.00E-04
	8	Leishmaniasis	2.80E-05	2.00E-04
	9	Type I diabetes mellitus	5.81E-05	4.00E-04
	10	Malaria	1.00E-04	6.00E-04
Gene Ontology	1	Response to molecule of bacterial origin	1.06E-06	0.0014
	2	Response to lipid	1.84E-05	0.0046
	3	Response to bacterium	2.76E-05	0.0046
	4	Regulation of cell proliferation	2.43E-05	0.0046
	5	Immune response	3.27E-05	0.0048
	6	Cell proliferation	4.70E-05	0.0053
	7	Cytokine activity	0.0003	0.0286

Appendix Figure S5. Grandin et al.

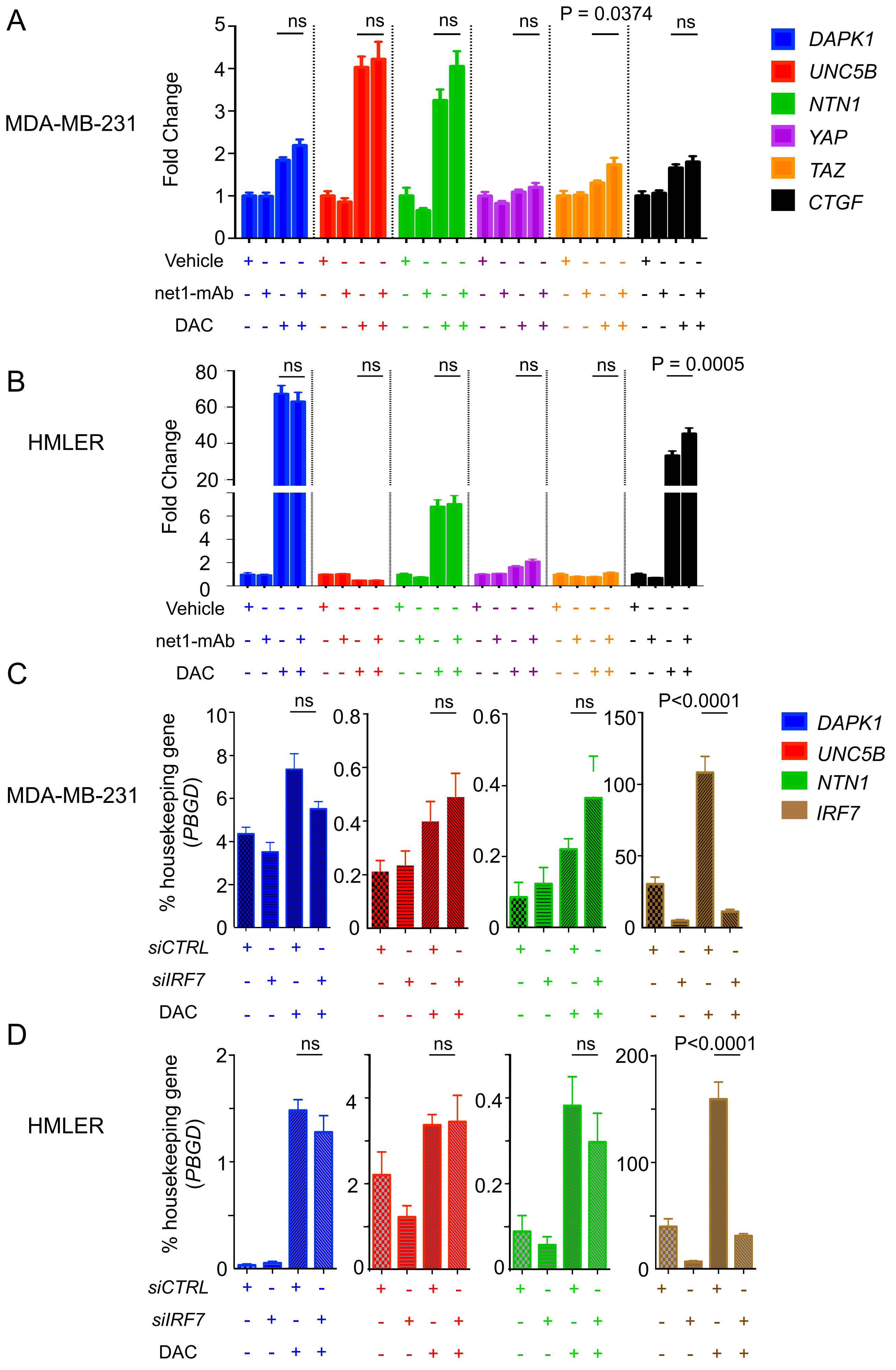

Appendix Figure S6. Grandin et al.

Appendix Table S1 - L ist of primers

**Q-RT-PCR Primers**	Forward (5' to 3')	Reverse (5' to 3')
PBGD	GAGTGATTCGCGTGGGTACC	GGCTCCGATGGTGAAGCC
GAPDH	CGGAGTCAACGGATTTGGTCGTAT	AGCCTTCTCCATGGTGGTGAAGAC
DAPK1	GATAGAAATGTCCCCAAACCTCG	TCTTCTTGGATCCTTGACCAGAA
UNC5B	CCCGCCACACAGATCTACTT	CAGTAATCCTCCAGCCCAAA
NTN1	TGCAAGAAGGACTATGCCGTC	GCTCGTGCCCTGCTTATACAC
MBD2	CCCACAACGAATGAATGAACAGC	TGAAGACCTTTGGGTAGTTCCA
UNC5A	ATCACCAAGGACACAAGGTTTGC	GGCTGGAAATTATCTTCTGCCGAA
UNC5C	GCAAATTGCTGGCTAAATATCAGGAA	GCTCCACTGTGTTCAGGCTAAATCTT
DCC	AGCCAATGGGAAAATTACTGCTTAC	AGGTTGAGATCCATGATTTGATGAG
YAP	CGCTCTTCAACGCCGTCA	AGTACTGGCCTGTCGGGAGT
TAZ	CCAGTGCCTCAGAGGTCCA	ATCTGCTGCTGGTGTTGGTG
CTGF	CCAATGACAACGCCTCCTG	TGGTGCAGCCAGAAAGCTC
IRF7	ACTGTGACACCCCCATCTTC	GCTGCTATCCAGGGAAGACACA
**Pyrosequencing**		
Amplification primers		
NTN1	BIO-TTATGGTTATTTATAAGTTTATGGA	TAACCCAATCCTACAACAC
DAPK1	BIO-TTTTTTGGATTGTGGAAATGTATAA	AACCCTAAACTACTACCTCTCCTCC
GAPDH unmodified	CTCTTGCTACTCTGCTCTGG	GCTAAGTTTAGCCTGCCTGG
GAPDH modified	GTATTTGTTGATGGGTTAAGG	ATAAAAACAAATCCCCTACCC
Sequencing primers		
NTN1-a	TAACCCAATCCTACAACAC	
NTN1-b	CAATCCTACAACAC	
DAPK1-a	AACCCTTAAATCAA	
DAPK1-b	TTCTCTATAATTTA	
**DNA methylation analysis**		
NTN1	ACACTGACGACATGGTTCTACATGAGTTGTGGTGAGTTTTTATTTT<br>TACGGTAGCAGAGACTTGGTCTAACAACATCCTTTAATCATCTTACAAC	
DAPK1	ACACTGACGACATGGTTCTACAGAGGTTTTTAGTGGATATGGGATT<br>TACGGTAGCAGAGACTTGGTCTTCCACCTCCAAAATTCAAATAATT	
**Human DNA analysis in Chicken lungs**		
Alu	CACCTGTAATCCCAGCACTTT	CCCAGGCTGGAGTGCAGT
Avian Repeat	ATAGAATGGCCTGGGTTGAAAAG	AAGTTTTTCACACAGAGGGTGGT
**siRNA**		
IRF7	CAGCCUCUAUGACGACAUC	
