# Supplementary material for: Inhibition of DNA methylation promotes breast tumor sensitivity to netrin‐1 interference
Source: EMBO Mol Med. 2016 Jul 4;8(8):863–77. doi: 10.15252/emmm.201505945 (PMC4967941; doi:10.15252/emmm.201505945)
Supplement: Supplementary file 2 — Expanded View Figures PDF [file EMMM-8-863-s002.pdf]

Expanded View Figures

Figure EV1. *NTN1* and *DAPK1* are concomitantly altered in human breast cancers.

A Bisulfite PCR sequencing (4 CpGs analyzed, region: light gray boxes in Fig 1A) indicated that DNA methylation of the *NTN1* CpG island (CGi) was inversely correlated with its expression. Pearson correlation, $P = 0.008$, $r = -0.55$, $n = 18$.

B Bisulfite PCR sequencing (3 CpGs analyzed, region: light gray boxes in Fig 2A) indicated that DNA methylation of the *DAPK1* CGi was inversely correlated with its expression. Pearson correlation, $P = 0.003$, $r = -0.66$, $n = 22$.

C Tissue microarrays (70 paraffin embedded sections) from human breast carcinomas were immuno-stained with antibodies against DAPK1, UNC5B, and netrin-1. Samples were classified in quartiles according to the level of netrin-1 expression. The levels of DAPK1 and UNC5B expression (index constructed from the percentage of sections exhibiting a positive staining) in the first and fourth quartiles of netrin-1 expressing groups were compared using a chi-squared test.

D Representative staining corresponding to low and high levels of expression is shown for each antibody, and expression levels were determined from the percentage of sections exhibiting a positive staining. As a control, staining of the samples using a non-related isotype antibody was performed.

E Quantification of the presence or absence of alterations in gene A was associated with the presence or absence of alterations in gene B in human breast tumors which was determined from cBioPortal web site, *z*-score threshold $\pm$ 2.2

F Individual methylation plots. Red rectangles represent methylated CpG and blue rectangles unmethylated CpG.

G Mean DNA methylation inhibition of *DAPK1* and *NTN1* in MDA-MB-231 and HMLER cells upon DAC treatment.

© 2016 The Authors

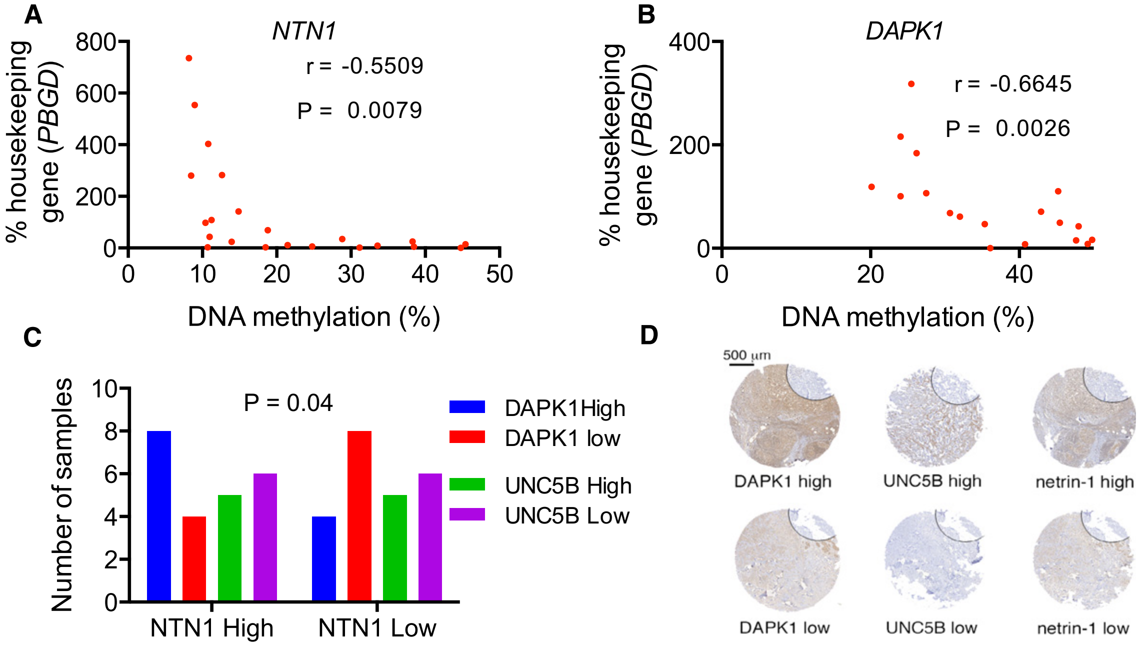

E

Gene A	Gene B	Odds Ratio	P value	Association
DAPK1	*NTN1*	4,71	0,003	Tendency towards co-occurrence
DAPK1	*UNC5B*	< -3	0,351	Tendency towards mutual exclusivity
UNC5B	*NTN1*	< -3	0.606	Tendency towards mutual exclusivity

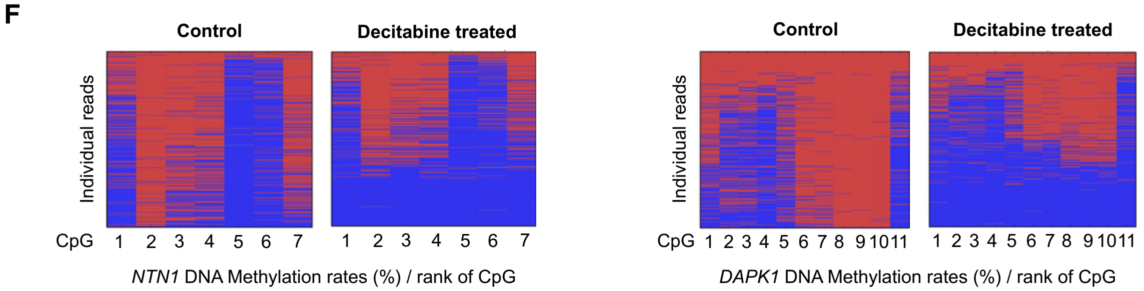

G

Cell line	Gene	DAC 10µM	Mean CpG methylation	Reads number	Mean % of inhibition
HMLER	*DAPK1*	No	0,92	1007	58%
			0,90	954	
		Yes	0,41	958	
			0,36	1406	
MDA-MB-231		No	0,70	1738	44%
			0,70	1499	
		Yes	0,38	2232	
			0,40	1930	
HMLER	*NTN1*	No	0,82	568	49%
			0,82	855	
		Yes	0,43	585	
			0,40	986	
MDA-MB-231		No	0,54	970	37%
			0,55	796	
		Yes	0,34	1225	
			0,34	1848	

	Start	End	Chromosome
Amplicon *DAPK1*	90111351	90112,05	9
Amplicon *NTN1*	8923810	8923810	17

Figure EV1.

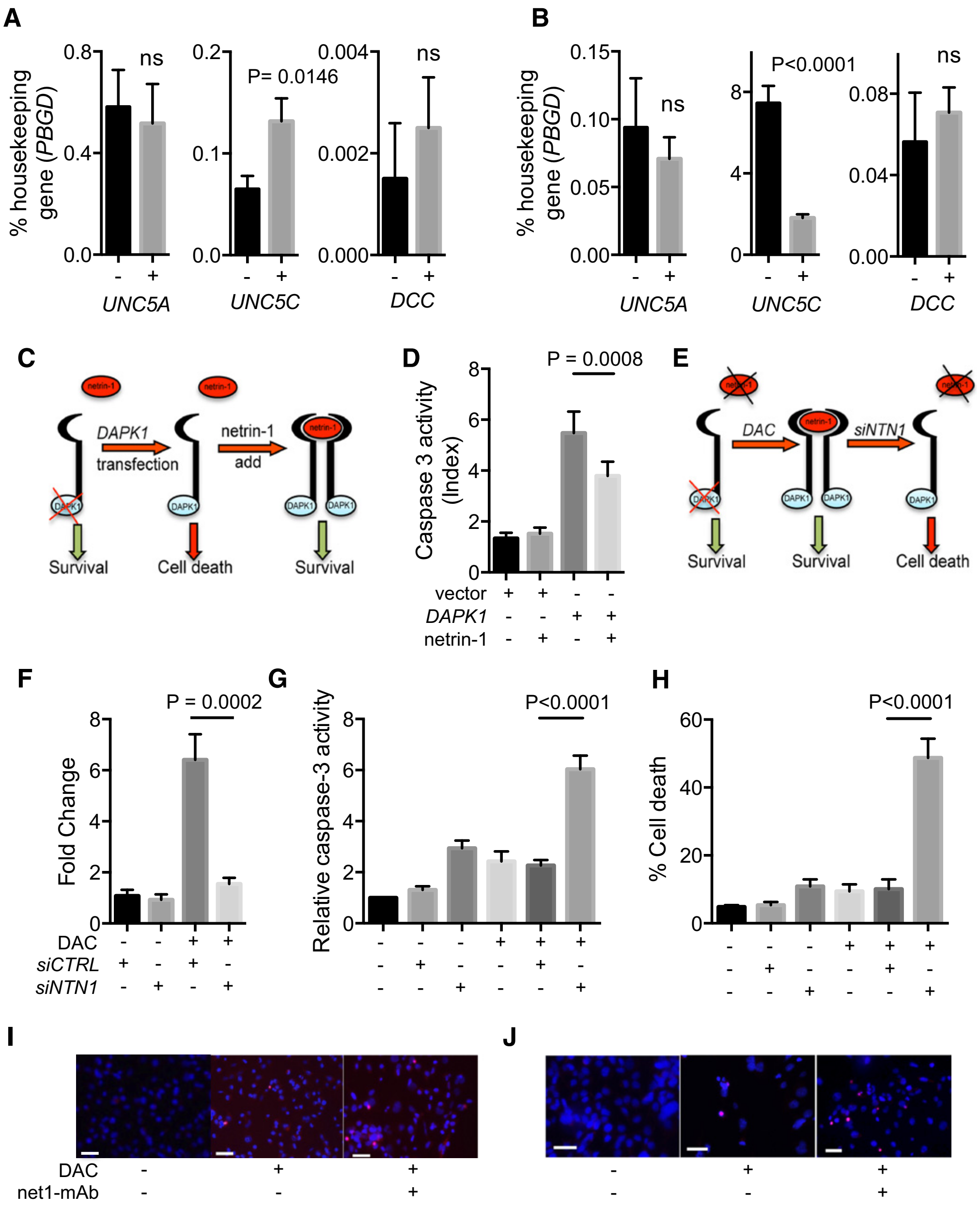

Figure EV2. The NTN1 DR apoptotic pathway, mechanism, and specificity.

A, B Other *NTN1* DR gene expression in breast cell lines, and impact of *DAPK1* and *NTN1* on the induction of apoptotic cell death *in vitro*. Gene expression was measured by qRT–PCR after 72 h in MDA-MB-231 (A) and HMLER (B) cells treated daily with 10 μM decitabine (DAC). The level of *PBGD* expression was used as an internal control. Data are represented as mean ± s.e.m. for to 3 independent experiments. ****$P < 0.0001$, two-tailed unpaired Student's *t*-test.

C Schematic representation of the cellular effect on HMLER cells of *DAPK1* overexpression and/or recombinant netrin-1 treatment.

D Caspase-3 activation upon *DAPK1* overexpression in HMLER and its reversion by the addition of netrin-1. ***$P < 0.0001$, one-way ANOVA.

E Schematic representation of DAC and effect of *NTN1* siRNA on HMLER cells.

F Effect of *NTN1* siRNA (si*NTN1)* on the expression of *NTN1* by qRT–PCR. The level of *PBGD* expression was used as an internal control. ***$P < 0.0001$, one-way ANOVA.

G, H si*NTN1* triggers apoptosis and cell death in hypomethylated HMLER cells. Cells were treated with DAC (10 μM, 72 h), and/or si*NTN1* (30 pmol, 48 h), and caspase-3 activity (G) and cellular mortality (H) were measured. As a control, a scramble siRNA was used. Data (G, H) are expressed as mean ± s.e.m. of at least 3 independent experiments. ****$P < 0.0001$, one-way ANOVA.

I, J Representative images of TUNEL experiments shown in Fig 3, in MDA-MB-231 and HMLER cells, respectively. Scale bars = 50 μm.

© 2016 The Authors

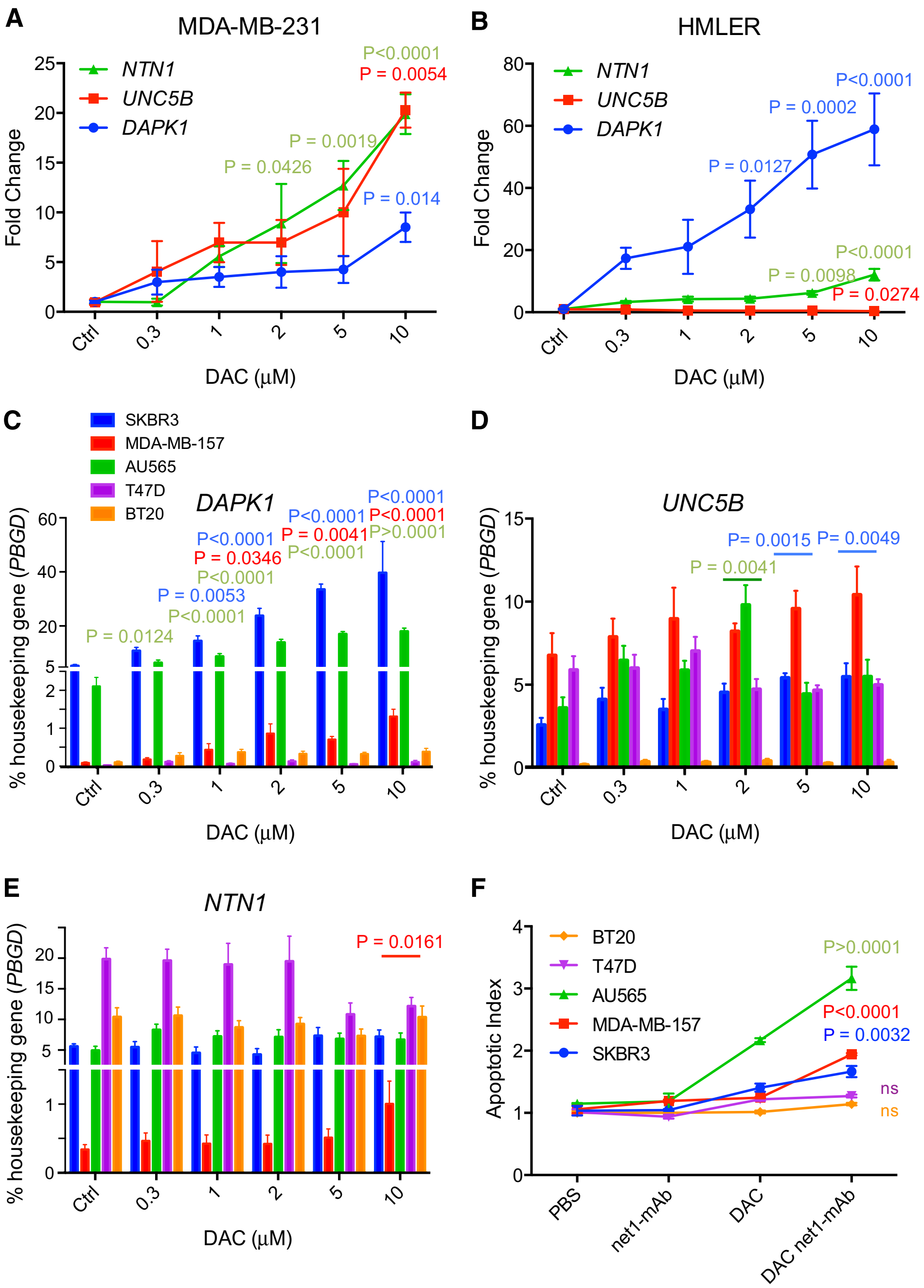

Figure EV3. Netrin-1 neutralizing antibody net1-mAb triggers apoptosis in DAC-treated breast cancer cell lines.

A–E Gene expression was measured by qRT–PCR after 72 h in human breast cancer cell lines: MDA-MB-231 (A), HMLER (B) and SKBR3, MDA-MB-157, AU565, T47D, and BT20 (C–E) treated daily with DAC at final concentrations of 0.3, 1, 2, 5, and 10 μM. The level of *PBGD* expression was used as an internal control. Data are expressed as mean ± s.e.m. of at least 3 independent experiments. ****$P < 0.0001$, one-way ANOVA. Colors of the stars correspond to the gene analyzed (A and B) or to the cell lines analyzed (C–F).

F Comparison of cellular mortality in DAC-treated cells versus DAC + net1-mAb treated cells assessed using the caspase cleavage assay. Data are expressed as mean ± s.e.m. of at least 3 independent. ****$P < 0.0001$, two-way ANOVA and *post hoc* Tukey test. ns = not significant.

© 2016 The Authors

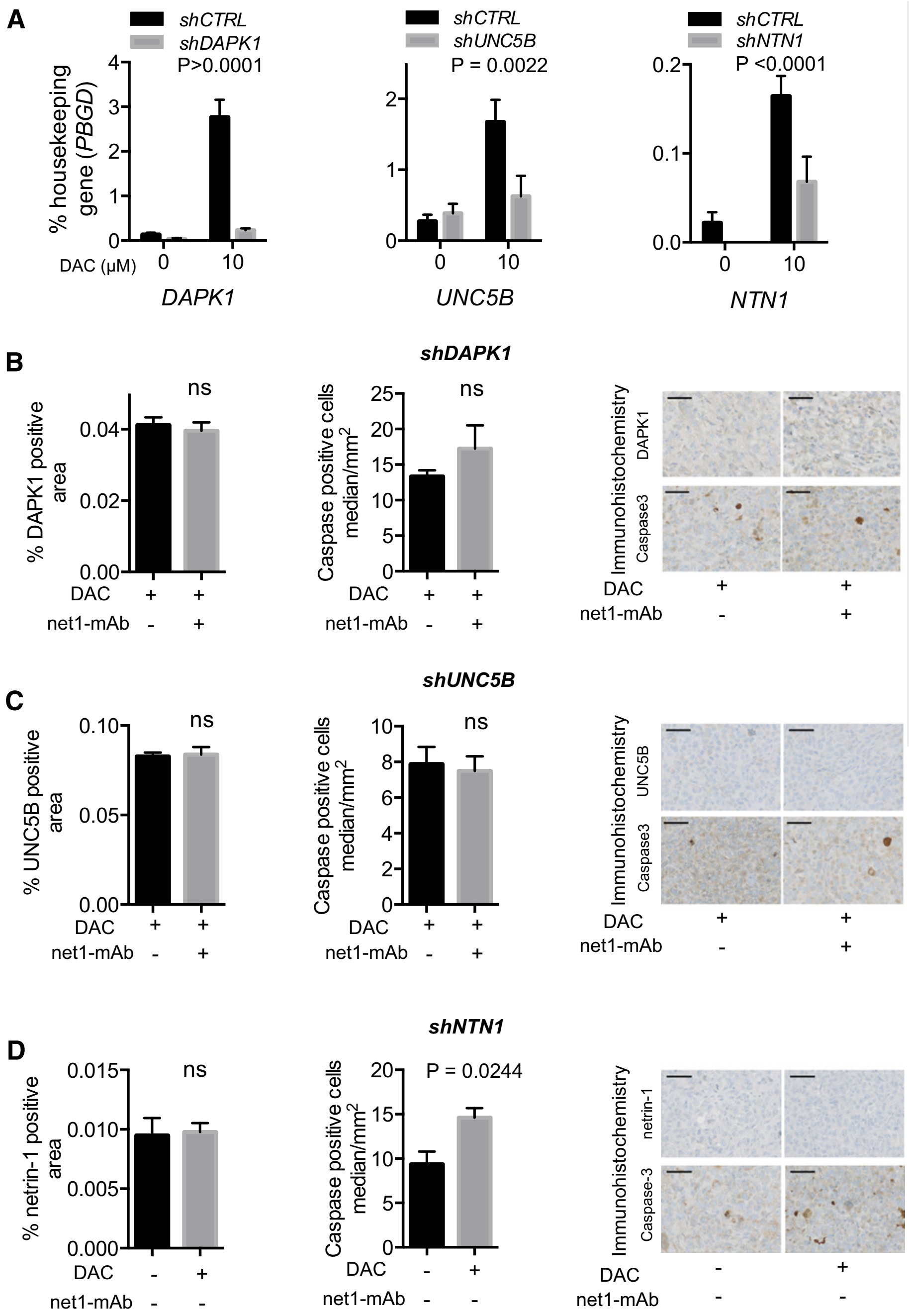

Figure EV4. Response of MDA-MB-231 cell lines stably transfected with shRNA targeting *DAPK1*, *UNC5B*, and *NTN1* to treatments combining DAC and net1-mAb.

A Gene expression was measured by qRT–PCR after 72 h in cell lines stably transfected with shRNA. Cells were treated daily with 10 µM DAC. The level of *PBGD* expression was used as an internal control. Data are expressed as mean ± s.e.m. of at least 3 independent experiments. ****$P < 0.0001$, one-way ANOVA.

B–D Levels of DAPK1, UNC5B, and netrin-1 were measured by immunohistochemistry in paraffin embedded xenografts of MDA-MB-231 stably transfected with shRNA. Left panels: levels of DAPK1 (B), UNC5B (C), and netrin-1 (D) in xenografts of MDA-MB-231 sh*DAPK1*, sh*UNC5B*, and sh*NTN1*, respectively. Protein levels are expressed as the percentage of total tumor surface; *P*-values from at least 3 tumors per group. Middle panels: median number of apoptotic cells per mm^2; values from at least 3 tumors per group. Error bars = s.e.m. Mann–Whitney test. ns = not significant. Right panels: representative sections of *in vivo* protein levels and induction of apoptosis in MDA-MB-231 tumors cells depleted of key genes from the netrin-1 dependence receptor pathway. Scale bars = 50 µm.

 © 2016 The Authors
